# Transfer of veterinary parasiticides from the fur lining bird’s nest to eggs and chicks

**DOI:** 10.1007/s11356-026-37654-7

**Published:** 2026-04-21

**Authors:** Cannelle Tassin- de-Montaigu, Gaetan Glauser, Sylvie Guinchard, Dave Goulson

**Affiliations:** 1 University of Sussex School of Life Sciences, Brighton, UK; 2 Faculte Des Sciences, Universite de Neuchatel, Neuchatel, Switzerland

**Keywords:** Fipronil, Imidacloprid, Pesticide, Flea, Great tit, Blue tit

## Abstract

Previously we have found that spot-on insecticides commonly applied to dogs and cats to deter ticks and fleas contaminate lining of great tit and blue tit nests via birds using fur to line their nests. Here we describe significant positive correlations between pesticide concentrations found in the nest lining fur and those in unhatched eggs and dead chicks from the same nests, indicating that these compounds transfer from contaminated nesting material into avian tissues. This study advances our understanding of how environmental contamination from domestic sources may directly impact wildlife health and development. It highlights the need for improved environmental risk assessment for veterinary products intended for use on companion animals, and more specifically the need to determine whether the exposure we identify lead to impacts on bird health in the short or long term.

**Supplementary Information:**

The online version contains supplementary material available at 10.1007/s11356-026-37654-7.

## Introduction

Since the 1960 s, the use of pesticides has been recognised as a major contributor to the decline of bird populations worldwide (Carson 1962; Mineau and Kern 2023; Rigal et al. 2023). While the risks from agricultural pesticide use are well-documented, the environmental impact of veterinary insecticides remains comparatively underexplored. Companion animals and livestock are routinely treated with parasiticides belonging to several chemical classes, including pyrethroids (e.g., permethrin, cypermethrin), macrocyclic lactones such as avermectins (e.g., ivermectin), neonicotinoids (e.g., imidacloprid) and phenylpyrazoles (e.g., fipronil). The latter two chemicals were both banned in agriculture in the EU and UK but are still authorised for veterinary use (European Commission, 2018a; European Commission, 2019; PAN UK, 2023; Preston-Allen et al. 2023). Environmental risk assessments for veterinary medicines used in non-food animals typically assume negligible environmental exposure. As a result, higher-tier ecotoxicological evaluations are rarely required prior to approval (EMA, 2000; 2012).

Imidacloprid and fipronil are widely used in veterinary medicine, often administered as topical spot-on treatments, sprays, and shampoos, and widely available to the public without prescription. Over 80% of cats and dogs in the UK receiving such treatments at least once per year, with the label recommending repeated application every 28 days (PDSA 2025). Veterinary sales data suggest that substantial quantities of these chemicals have entered domestic environments over the past decades (VMD 2025a, b). Following application, both compounds persist on animal fur for extended periods, frequently beyond the recommended treatment interval (Perkins et al. 2025), and can be transferred to the surrounding environment through direct contact, shedding, bathing, swimming, and household contamination (Craig et al. 2005; Dyk et al. 2012; Perkins et al. 2021; 2024; 2025; Yoder et al. 2024). Indeed, recent studies have found widespread contamination of English rivers, with 99% of samples containing one or both chemicals and chronic toxicity limits often far exceeded (Perkins et al. 2021). The continuous release from numerous treated animals ensures the consistent presence of these pesticides in aquatic ecosystems (Yamamuro et al. 2019; Diepens et al. 2023). Given their potency, fipronil, imidacloprid and subsequent metabolites have been associated with oxidative stress, endocrine disruption, and adverse reproductive effects in birds and other vertebrates (Lopez-Antia et al. 2015a, b; Merga and Van den Brink 2021; Addy-Orduna et al. 2024), making them relevant candidates for investigating contaminant transfer from nest materials to avian eggs and chicks (Anadón et al. 2025).

Previous research has shown that many bird species, including cavity-nesting passerines, commonly collect animal fur to line their nests (Collias and Collias 2014; Harničárová and Adamík 2016), a behaviour that may unintentionally expose birds to veterinary insecticides when fur originates from treated pets. In an earlier study, insecticide residues were detected in the fur used as nesting material in blue tit (*Cyanistes caeruleus*) and great tit (*Parus major*) nests. One hundred per cent of nests tested contained the compound fipronil (Tassin de Montaigu et al. 2025). While that study documented the prevalence of these compounds and examined their potential associations with mortality of the bird’s offspring (e.g., number of unhatched eggs and dead chick in nests), direct evidence of contaminant transfer from the nest materials to avian tissues remained lacking.

Here, we target insecticides used in veterinary medicine or detected as legacy contaminants in animal-derived materials (see the complete list of compounds and their chemical structures, molecular formulae, molecular weights, and CAS numbers in Supplementary Material S3). Target compounds include neonicotinoids (e.g. imidacloprid, acetamiprid) and some of their metabolites (e.g. desnitro-imidacloprid, imidacloprid-olefin, acetamiprid-desmethyl), pyrethroids (e.g., permethrin, deltamethrin and cypermethrin), phenylpyrazoles (fipronil, its major metabolites fipronil sulfone and fipronil sulfide), and one organophosphate (chlorpyrifos) historically used as an ectoparasiticide (up until 2020). Each compound has a specific mode of action and metabolism, for instance, neonicotinoids act as agonists of nicotinic acetylcholine receptors and undergo metabolic conversion in vertebrates to several metabolites which may retain biological activity and environmental persistence (Wang et al. 2018). Fipronil acts as a GABA chloride channel antagonist and is extensively metabolised following topical application, primarily to fipronil sulfone through oxidative processes, with additional minor metabolites such as fipronil sulfide (Wang et al. 2016). However, little is known about their accumulation or degradation in animal hair. However, concentrations of fipronil were found to be on average at 82 µg/g (range 34–15) around the neck of dogs 4 weeks after being treated with Frontline® (Dyk et al. 2012).

Here we build upon this previous work by testing unhatched eggs and dead chicks for pesticide residues. We test the hypothesis that these pesticide residues originate from the nest lining fur by testing whether concentrations in the unhatched eggs and dead chicks are predicted by concentrations in nest lining. By focusing on compounds of high concern, particularly fipronil and imidacloprid, we aim to provide evidence of environment-to-organism transfer of veterinary insecticides in wild birds. This study not only highlights a previously underappreciated exposure route but also raises questions about the broader ecological risks posed by common veterinary treatments.

## Methods

This study uses the same nests as Tassin de Montaigu et al. (2025), and while the first study goal was a general screening of insecticides, we used the same methods for fur sample preparation, insecticide selection, and chemical analysis (including sample extraction and sample analysis for fur samples). Therefore, a shortened version of the methods is given here, see Tassin de Montaigu et al. (2025) for a more thorough description.

### Sample collection and preparation

Nests were collected across the United Kingdom (UK) in autumn 2020 by volunteers from the British Trust for Ornithology (BTO) “Nesting Neighbours” scheme and via the social platform “X” (called “Twitter” at the time of the study), using detailed collection guidelines (Supplementary information S1–S2).

A total of 237 nests (168 blue tit, 69 great tit) were received, including information on species, habitat type (urban/rural), and numbers of unhatched eggs or dead chicks. When no data were reported, missing values were recorded. Due to natural nest-cleaning behaviour by adults, actual numbers of eggs/chicks may be underestimated. Volunteers also reported pet ownership, flea/tick treatments, and nearby livestock presence (within 200 m, which covers both species’ territory sizes (supplementary information S1–S2).

Nests were processed by collecting fur used as the inner lining, unhatched eggs, and dead chicks, cleaning any tools and the work surface between each nest using acetone to avoid cross-contamination. The total fur collected from each nest was mixed to homogenise the potential nest concentration; and the unhatched eggs and dead chicks were first dehydrated completely (35 °C, LEEC® drying cabinet) and then all unhatched eggs or dead chicks from the same nest were grouped together to provide a measure of mean offspring contamination and increase the numbers of useable samples. The various decomposition stages (e.g., mummified for dead chicks) of unhatched eggs and dead chicks did not allow for them to be washed prior to sample preparation, nor did it allow us to determine the development stages of the chicks nor the separation of specific tissues (e.g., liver). A total of twenty-five mg (± 2.5 mg) of fur (finely cut), eggs (whole egg powdered with pestle and mortar) and chicks (whole chick mixed using a blender) were needed for the analysis, to enable accurate quantification of insecticide residues. Final usable samples included 103 nests (64 blue tit nests and 39 great tit nests) with fur samples, 24 nests with unhatched eggs (15 blue tit nests and 9 great tit nests), and 35 nests with dead chicks (20 blue tit nests and 15 great tit nest) out of which 62.1%, 62.5%, and 65.7% were from rural areas and 37.9%, 37.5%, and 34.3% from urban areas for fur, eggs and chicks respectively.

Second broods for blue and great tits are rare in the UK, so nests were assumed to have been used only once (Sussex Wildlife Trust,  n.d.).

#### Insecticide selection

Fifteen insecticides (including 8 common ectoparasiticides: imidacloprid, fipronil, dinotefuran, nitenpyram, permethrin, cypermethrin, chlorpyrifos, and deltamethrin, and 2 compounds used as both ecto- and endoparasiticides: ivermectin and thiacloprid) and five of their metabolites (imidacloprid-olefin, desnitroimidacloprid, fipronil sulfide, fipronil sulfone, and desmethyl-acetamiprid) were selected based on known UK usage patterns and analytical method availability at Neuchâtel’s chemistry platform. The selected neonicotinoids represent different chemical generations, encompassing compounds with contrasting receptor affinity, environmental persistence, and metabolic fate, thereby capturing a broad range of environmentally relevant exposure scenarios. These insecticides were further selected because their toxicological mechanisms are well characterised and include induction of oxidative stress, disruption of detoxification pathways, and alteration of cellular energy metabolism, processes increasingly linked to sublethal developmental and reproductive effects in vertebrates (Wang et al. 2018). Pyrethroids (e.g., permethrin) and macrocyclic lactones (e.g., ivermectin) were additionally included due to their widespread veterinary use as authorised ectoparasiticides and endoparasiticides within the European Union, where topical applications to companion animals and livestock represent an important pathway for secondary environmental contamination (Anadón et al. 2025). Fipronil and its principal metabolites were specifically incorporated owing to their environmental persistence and bioactivation into toxic derivatives, such as fipronil sulfone, which substantially contribute to chronic toxicity and oxidative stress responses in non-target organisms (Wang et al. 2016). Some additional neonicotinoids and metabolites were included due to existing laboratory protocols at the testing facility (Neuchâtel Platform of Analytical Chemistry, Faculty of Sciences, University of Neuchâtel; Supplementary Information S3).

### Chemicals and sample analysis

For chemical analysis, sample extraction and sample analysis protocol and method see Tassin de Montaigu et al. (2025). While the previous study only references the protocol and method for the fur samples, the exact same method has been applied to the unhatched egg and dead chick samples. Insecticide residues were quantified using UHPLC-MS/MS on an Acquity UPLC I-Class system coupled to a TQ-XS triple quadrupole mass spectrometer (Waters). Separation was achieved on a Kinetex PFP column (75 × 2.1 mm, 2.6 µm) using water (0.05% acetic acid, 1 mM ammonium acetate) and methanol as mobile phases. Detection was performed in multiple reaction monitoring (MRM) mode with fast polarity switching. Samples were extracted using a modifies QuEChERS protocol and quantified using isotopically labelled internal standards.

The method demonstrated adequate specificity and selectivity based on compound-specific MRM transitions and retention times. Linearity was confirmed using six-point calibration curves (0.01–20 ng/mL). Precision showed coefficients of variation below 15%, and accuracy was verified for the fur by recovery experiments (80–120% at 10 ng/g, *n* = 5). The limit of detection (LODs) for the method ranged from 0.03 to 2.67 ng/g of fur, egg, chick, while the limits of quantification (LOQs) ranged between 0.08 and 8 ng/g. The specific LOD and LOQ for each insecticide is given in supplementary information S3. Robustness and system suitability were ensured through regular analysis of blanks and quality-control standards throughout each batch.

### Statistical analysis

To explore the potential for transfer of veterinary insecticides from nest lining to offspring (egg and chick), we used the following variables: the total concentration of insecticides (defined as the sum concentration of all insecticides, including metabolites, found per nest), the total number of insecticides (defined as the total number of insecticides, including metabolites, found per nest), and specific insecticides concentrations (fipronil and imidacloprid along with their respective metabolites fipronil sulfone, fipronil sulfide, imidacloprid-olefin, desnitro-imidacloprid). The concentration of fipronil, imidacloprid and metabolites, and total concentration of insecticides, included: the concentrations found when > LOQ and for the concentrations LOQ > x > LOD, the LOD value was taken, as a conservative approach. The two bird species were pooled to maximise statistical power for analyses.

For the statistical analyses we first identified and removed outliers for the unhatched eggs, dead chick and fur dataset for each of the variables of interests (the total concentration of insecticides, the total number of insecticides and the various specific insecticides concentrations), using the InterQuartile Range (IQR) method. For the unhatched egg data, the number of outliers found ranged between 3 and 4; for the dead chicks, it ranged between 2 and 7 outliers, and for the fur it ranged between 7 and 17. In addition, we performed a visual inspection together with a statistical comparisons of geographic coordinates (*t*-test) of the spatial distribution of the respective sample (unhatched eggs, dead chicks, and fur samples) and found no evidence that the total concentration of insecticides were spatially clustered, suggesting that extreme values were not associated with specific geographic areas.

A paired *t*-test was performed to compare the average concentration of insecticides found between fur samples and unhatched eggs and between fur samples and dead chicks. Next, the concentrations found in fur, eggs, and chicks for all variables were z-score standardised (z = (x – mean)/standard deviation) due to the high difference in scale between fur, egg, and chick concentrations. Finally, after testing for normality (Shapiro test), we performed correlation tests (Pearson when normally distributed or Spearman when not normally distributed) between the insecticide concentrations found in fur and eggs (total insecticide concentration, total number of insecticides found, concentrations of specific insecticides fipronil, imidacloprid and metabolites) and between fur and chicks.

The sample size to compare the insecticide levels between egg and chick from the same nest was too small (*n* = 4) to be of any statistical relevance, though the figures are available in Table 3. After having removed the outliers, the samples size for fipronil sulfide was also too small to be used. Similarly, we could not compare the concentrations found between habitat type (urban: *n* = 8, rural: *n* = 12). All statistical analyses were performed using R (v 4.5.0, R Core Team 2025).

## Results

Out of the 103 nests received and analysed in the previous study, a total of 24 nests had at least one unhatched egg (62.5% from blue tit nests and 37.5% from great tit nests), and 35 nests had at least one dead chick (57.1% from blue tit nests and 42.9% from great tit nests). Among volunteers contributing nests, 54.4% reported owning at least one dog or cat, and 83.9% of these pets were treated with ectoparasitic products such as spot-on formulations, collars, or oral medications.

### Insecticide residues found in eggs and chicks

In unhatched eggs, 13 insecticides were found out of the 20 we screened for. The number of insecticides ranged from a minimum of 0 to a maximum of 7 compounds in a single sample with an average of 2.29. The total concentration of insecticide found in a single egg sample ranged from 0.05 to 133.07 ng/g with an average of 22.48 ng/g. In dead chicks, 15 different insecticides were found, ranging from 0 to up to 8 insecticides in a single dead chick (average 3.17 per chick) and the total concentration of insecticides ranges from 0.53 to 1098 ng/g with an average of 168 ng/g. In the fur, 17 insecticides were found and the total concentration range between 0.83 ng/g and 11,043 ng/g (see Tassin de Montaigu et al. 2025).

In unhatched egg samples, fipronil sulfone (66.67% in blue tit and 55.56% in great tit), fipronil (40% in blue tit and 33.33% in great tit) and imidacloprid (40% in blue tit and 22.22% in great tit) were the active substances found at the highest percentage of samples. In addition, permethrin, cypermethrin and desnitro-imidacloprid were also found in 22.22% of the eggs from great tit nests (Table 1). The highest concentration of any one pesticide was of cypermethrin in a great tit egg sample (123.42 ng/g), followed by fipronil (65.82 ng/g) and imidacloprid 60.38 ng/g) in blue tit egg samples (Table 1). Less frequently detected compounds included the maternal thiacloprid, acetamiprid, deltamethrin, chlorpyrifos and dinotefuran and the metabolites fipronil sulfide and imidacloprid-olefin. There were differences observed between each species, with some compounds like thiacloprid, acetamiprid, deltamethrin, and dinotefuran only being detected in blue tit eggs; cypermethrin and fipronil sulfide only being detected in great tit eggs.

**Table 1:** Summary statistics. Insecticides found in the unhatched eggs of blue tit and great tit, alongside their corresponding percentage of samples with concentration over the Limit Of Detection (LOD), maximum concentration (max CO), median concentration (median CO), average concentration (average CO) and their corresponding Standard Error concentration (SECO), all given in ng/g)

Insecticide residues found in unhatched eggs										
	Blue tit (*N* = 15)					Great tit (*N* = 9)				
Compound	*N*	% > LOD	max CO	median CO	Average CO (± SE)	*N*	% > LOD	max CO	median CO	Average CO (± SE)
Fipronil	6	40	65.83	4.07	14.07 (10.37)	2	22.22	55.92	28.97	28.97 (26.95)
Fipronil sulfone	10	66.67	24.56	1.44	4.62 (2.41)	5	55.56	40.35	1.25	8.97 (7.85)
Fipronil sulfide	0	-	-	-	-	1	11.11	1.46	-	-
Imidacloprid	6	40	60.38	2.41	11.81 (9.72)	3	33.33	15.53	12.19	9.82 (4.15)
Desnitro-imidacloprid	1	6.67	1.33	-	-	2	22.22	1.57	1.08	1.08 (0.5)
Imidacloprid-olefin	1	6.67	3.99	-	-	1	11.11	3.07	-	-
Permethrin	1	6.67	19.73	-	-	2	22.22	15.17	8.2	8.2 (6.98)
Thiacloprid	1	6.67	0.13	-	-	0	-	-	-	-
Acetamiprid	2	13.33	0.24	0.22	0.22 (0.03)	0	-	-	-	-
Cypermethrin	0			-	-	2	22.22	123.42	66.77	66.77 (56.66)
Deltamethrin	1	6.67	17.16	-	-	0	-	-	-	-
Dinotefuran	1	6.67	-	-	-	0	-	-	-	-

*N*: sample size; *%* > *LOD*: percentage of samples with a concentration above the LOD; *max CO*: maximum concentration (ng/g); *median CO*: median concentration (ng/g); *average CO*: average concentration ± Standard Error (ng/g)

For dead chicks, we found similar results with fipronil sulfone (45% in blue tit and 66.67% in great tit), fipronil (40% in blue tit and 40% in great tit) and imidacloprid (50% in blue tit and none in great tit) being the most frequently found in the highest percentage of samples. Cypermethrin was also found in 40% of blue tit chick samples being above the LOD (Table 2). Similarly, cypermethrin (1012.38 ng/g), fipronil (452.17 ng/g) and imidacloprid (451.86 ng/g) were the insecticides found at the highest concentrations across all chick samples (Table 2). Once again, some disparity was observed between species, with blue tit chick samples containing imidacloprid, thiacloprid, ivermectin, dinotefuran, and desmethyl-acetamiprid while these were absent in great tit chick samples.

**Table 2:** Summary statistics. Insecticides with traces found in the dead chicks of blue tit and great tit, alongside the corresponding percentage of samples with concentration over the Limit Of Detection (LOD), maximum concentration (max CO), median concentration (median CO), average concentration (average CO) and their corresponding Standard Error concentration (SE CO), all given in ng/g

Insecticide residues found in dead chicks											
	Blue tit (*N* = 20)					Great tit (*N* = 15)					
Compound	N	% > LOD	max CO	median CO	average CO (± SE)		N	% > LOD	max CO	median CO	average CO (± SE)
Fipronil	8	40	142.09	13.37	41.73 (20.8)		6	40	452.17	40.46	121.85 (71.9)
Fipronil sulfone	9	45	33.48	6.57	9.89 (4.04)		10	66.67	87.21	4.46	14.8 (8.44)
Fipronil sulfide	2	10	5.63	3.71	3.71 (1.92)		4	26.67	3.96	2.4	2.38 (0.77)
Imidacloprid	10	50	451.86	45.44	100. (45.75)		0	-	-	-	-
Desnitro-imidacloprid	3	15	5.95	3.73	4.45 (0.75)		3	20	6.34	5.83	4.75 (1.35)
Imidacloprid-olefin	7	35	115.53	9.93	30.19 (15.75)		5	33.33	50.61	12.46	21.87 (8.48)
Permethrin	6	30	322.28	18.13	68.82 (51.08)		4	26.67	34.75	6.4	12.55 (7.59)
Thiacloprid	2	10	1.27	0.79	0.79 (0.49)		0	-	-	-	-
Cypermethrin	8	40	310.91	19.11	54.43 (36.81)		2	13.33	1012.38	507.99	507.99 (504.4)
Deltamethrin	1	5	9.71	-	-		1	6.67	3.87	-	-
Ivermectin	1	5	142.41	-	-		0	-	-	-	-
Dinotefuran	1	5	305.52	-	-		0	-	-	-	-
Desmethyl-acetamiprid	1	5	4.65	-	-		0	-	-	-	-

*N*: samples size; *%* > *LOD*: percentage of samples with a concentration above the LOD; *max CO*: maximum concentration (ng/g); *median CO*: median concentration (ng/g); *average CO*: average concentration ± Standard Error (ng/g)

### Correlations between contamination of nest fur versus eggs and chicks

The insecticide residues detected across nest fur, unhatched eggs, and dead chicks, showed significant differences in mean concentrations between matrices. The mean total concentration of insecticides was significantly higher in nest fur (399.04 ± 114.28 ng/g) than in unhatched eggs (4.5 ± 1.39; t(17) = 3.46, *p* < 0.05) and significantly higher in nest fur (775.94 ± 145.66) than dead chicks (124.24 ± 31.34; t(31) = 5.35, *p* < 0.001).

### Fur versus unhatched egg samples

**Fig. 1:**
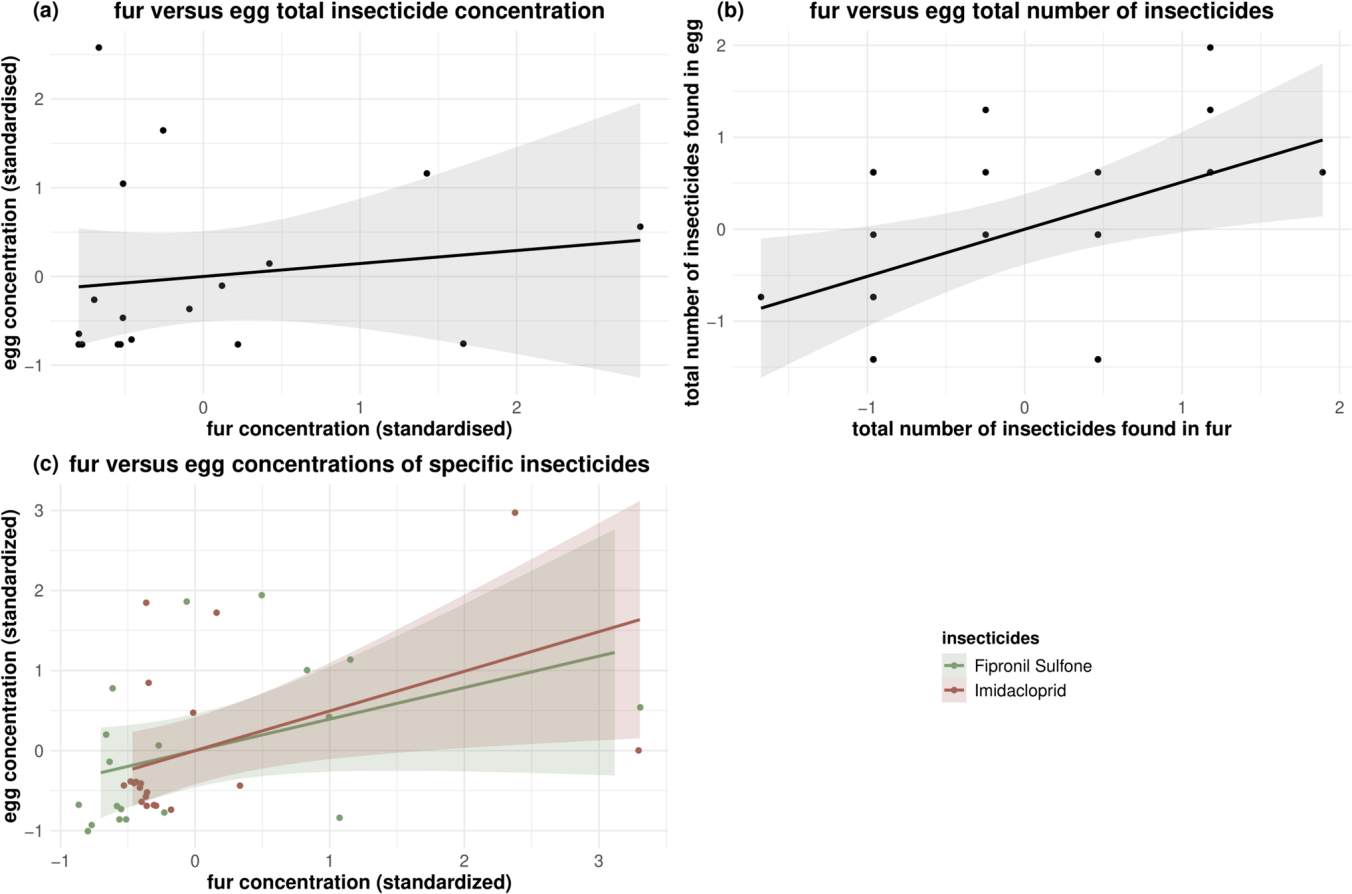
Insecticide correlations between fur and unhatched eggs. (**a**) The total insecticide concentration, (**b**) the total number of insecticides, and (**c**) the concentration of specific compounds (fipronil sulfone, and imidacloprid) found in the fur lining versus unhatched eggs. The shaded band shows the 95% confidence interval of the fitted values. For visual clarity in (**a**) and (**b**), the points are jittered

The total number of insecticides detected in fur was significantly correlated with the concentrations found in eggs (ρ = 0.53, *p* < 0.01, Table 3, Fig. 1b), but there was no significant correlation between the total concentration of insecticides in fur versus egg samples (Fig. 1a).

**Table 3:** Summary of the correlation tests (Spearman or Pearson) looking at the relationship between the fur and unhatched egg samples and between fur and dead chick samples, for the total insecticide concentration, total number of insecticides, imidacloprid and its metabolites imidacloprid olefin and desnitro imidacloprid, and for fipronil and its metabolite fipronil sulfone. Both species (blue and great tit) are pooled together. SE: standard-error, in bold are the *p*-values above the 0.05 threshold

	Correlation test (Spearman or Pearson)					
	Insecticide concentration found in eggs (z-std)			Insecticide concentration found in chick (z-std)		
Insecticide concentration found in eggs (z-std)				Variable	*t* or S value (df or rho)	*p* value
				Total insecticide concentration	*t* = 26.48 (df = 1)	*p* < 0.05
				Total number of insecticides	*t* = 1.96 (df = 1)	*p* > 0.05
				Imidacloprid	*t* = 10.81 (df = 2)	*p* < 0.01
				Imidacloprid olefin	-	-
				Desnitro imidacloprid	-	-
				Fipronil	*t* = 0.69 (df = 2)	*p* > 0.05
				Fipronil sulfone	*t* = 2.47 (df = 2)	*p* > 0.05
Insecticide concentration found in fur (z-std)	Variable	*t* or *S* value (df or rho)	*p* value	Variable	*t* or *S* value (df or rho)	*p* value
	Total concentration insecticide	*S* = 638.57 (rho = 0.34)	*p* > 0.05	Total insecticide concentration	*S* = 790.57 (rho = 0.86)	*p* < 0.001
	Total number of insecticides	*S* = 945.7 (rho = 0.53)	*p* < 0.01	Total number of insecticides	*S* = 1741.2 (rho = 0.61)	*p* < 0.001
	Imidacloprid	*S* = 409.04 (rho = 0.69)	*p* < 0.001	Imidacloprid	*S* = 757.78 (rho = 0.74)	*p* < 0.001
	Imidacloprid olefin	-	-	Imidacloprid olefin	*S* = 1148.7 (rho = 0.5)	*p* < 0.05
	Desnitro imidacloprid	-	-	Desnitro imidacloprid	-	-
	Fipronil	*S* = 958.4 (rho = 0.28)	*p* > 0.05	Fipronil	*S* = 1237 (rho = 0.62)	*p* < 0.001
	Fipronil sulfone	*S* = 458.97 (rho = 0.59)	*p* < 0.01	Fipronil sulfone	*S* = 1227.8 (rho 0.63)	*p* < 0.001

At the compound level, imidacloprid concentrations in fur were positively correlated with those in eggs (ρ = 0.69, *p* < 0.001, Table 3, Fig. 1c). In addition, while there was no significant correlation found for fipronil (ρ = 0.28, *p* > 0.05, Table 3), its metabolite, fipronil sulfone concentrations was also found to be positively correlated between fur and egg samples (ρ = 0.59, *p* < 0.01, Table 3, Fig. 1c).

### Fur versus dead chick samples

**Fig. 2:**
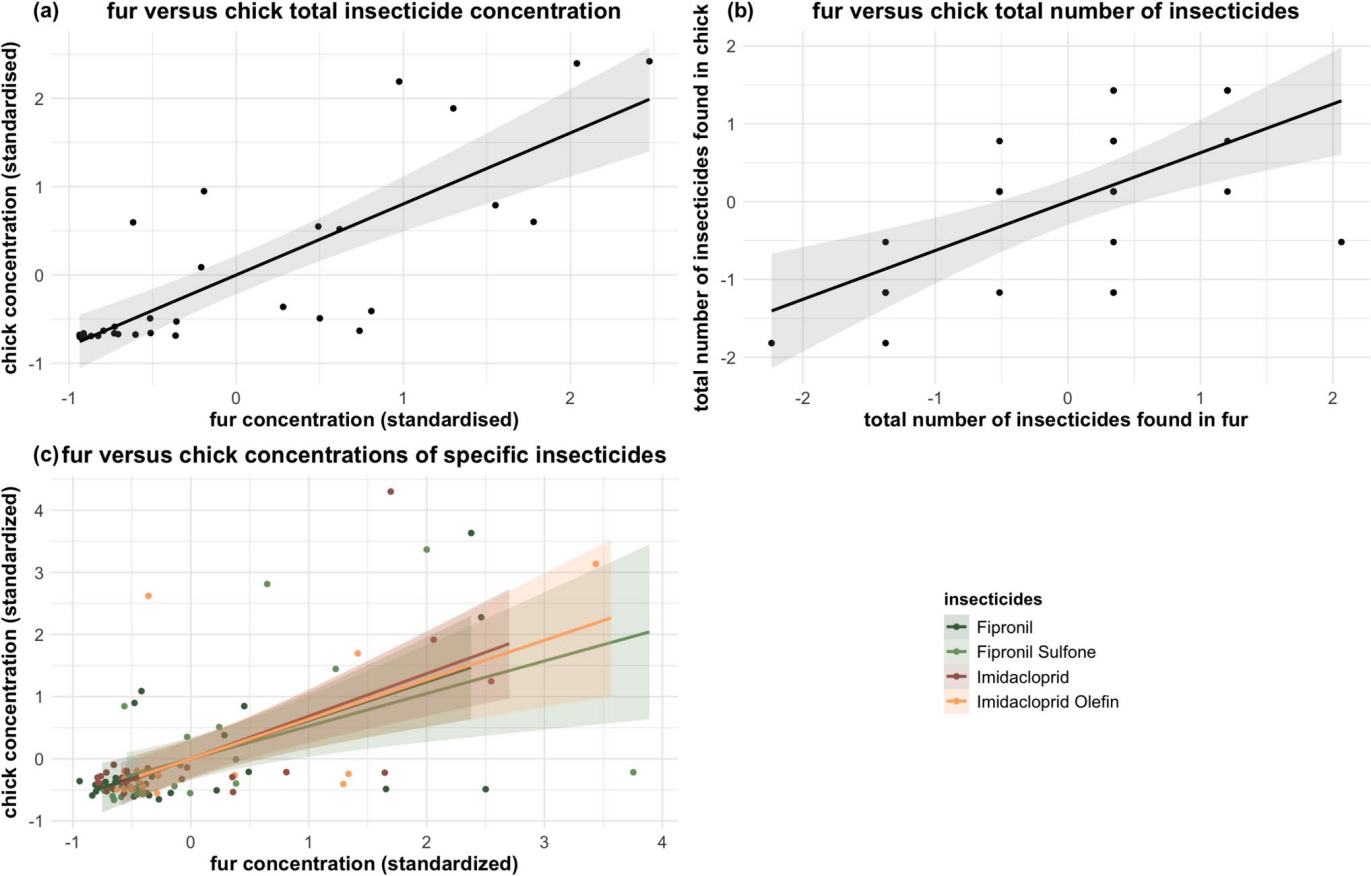
Insecticide correlation between fur and dead chicks. (**a**) The total insecticide concentration, (**b**) the total number of insecticides, and (**c**) the concentration of specific compounds (fipronil, fipronil sulfone, imidacloprid, and imidacloprid olefin) found in the fur lining versus the dead chicks. The shaded band shows the 95% confidence interval of the fitted values. For visual clarity in (**a**) and (**b**), the points are jittered

Dead chicks showed stronger correlations with insecticide residues detected in nest fur, likely due to an overall bigger sample size. The total concentration of insecticides in chicks was highly correlated with fur levels (ρ = 0.86, *p* < 0.001, Table 3, Fig. 2a), and so was the total number of insecticides (ρ = 0.61, *p* < 0.001, Table 3, Fig. 2b).

At the compound level, imidacloprid residues in chicks were strongly correlated with fur concentrations (ρ = 0.74, *p* < 0.001, Table 3, Fig. 2c), as were its metabolite imidacloprid olefin (ρ = 0.50, *p* < 0.05, Table 3, Fig. 2c). The concentration of fipronil itself was also significantly correlated between chicks and fur (ρ = 0.62, *p* < 0.001, Table 3, Fig. 2c) as were its metabolite fipronil sulfone (ρ = 0.63, *p* < 0.001, Table 3, Fig. 2c).

## Discussion

Our results reveal strong and significant positive correlations between insecticide concentrations, particularly imidacloprid, fipronil and their metabolites imidacloprid olefin and fipronil sulfone, found in the nest lining fur, and those in the unhatched eggs and dead chicks, suggesting that these compounds can move from contaminated nesting material into avian tissues. These results demonstrate that fur contamination acts as a predictor of egg and chick exposure, highlighting nest material as a route of transfer during development. This represents some of the first evidence of such transfer in wild bird populations, raising urgent questions about the broader ecological risks associated with veterinary parasiticide use. While the sample size of nests with both unhatched eggs and dead chick did not allow for robust statistical analysis, the insecticide residues found in chicks seems higher than the residues found in eggs. Perhaps this can be explained by maternal transfer, a difference in eggshell versus skin absorption, and/or, while dead chicks found in the nests were of seemingly different development stages, chicks are able to move in the nest and are more likely to ingest some of the compounds. Therefore, while the lining of nests lined with treated pet fur provides a potential direct dermal and ingestion route, the systemic nature and environmental persistence of fipronil and imidacloprid (Pisa et al. 2015) suggest that these molecules may also accumulate in the invertebrate prey (e.g., Lepidoptera larvae) consumed by blue and great tit and/or drinking water, potentially compounding the toxicological burden through ingestion.

While the concentrations found in eggs and chicks cannot be directly correlated with known levels from pet ectoparasitic treatments, the fipronil concentration at a dog’s application site (the neck) four weeks after treatment is 1000 times higher than what was detected in the nest fur (Dyk et al. 2012). This suggests that fur used in the nests, even if from a treated pet, likely did not originate from the application area. It could also indicate a drastic decrease in concentration during the six to seven months between nest construction and sample collection.

Fipronil and imidacloprid are potent neurotoxic insecticides originally developed for crop protection in the 1980 s and 1990 s respectively (Tingle et al. 2003; Krämer and Mencke 2012; Pisa et al. 2015) but no-longer authorized agricultural settings across the EU and UK due to their detrimental effects on non-target organisms, particularly pollinators (European Commission 2013, 2018b). Despite these bans, they remain widely used in the veterinary sector for spot-on treatments of domestic cats and dogs. We found that 83% of our volunteers’ pets received ectoparasitic treatment (also see Tassin de Montaigu et al. 2025), and similar number were found in a previous study with around 86.1% of dogs and 91.1% of cats owners administered treatments in the last 12 month prior to their survey (Perkins and Goulson 2023). While newer classes of insecticides used as ectoparasitic treatment have been introduced on the market (e.g., isoxazolines) sales data for imidacloprid and fipronil still contributes to a major part of the market in the UK with respectively 1,850,000 and 1,680,000 estimated number of doses delivered (Wells & Collins 2022; Cooper et al. 2020).

Their chemical properties, being water soluble and persistent, mean they have high potential for environment contamination. Recent studies have demonstrated their presence in freshwater systems throughout the UK, often at concentrations exceeding ecotoxicological thresholds for aquatic invertebrates (Hayasaka et al. 2012; Van Dijk et al. 2013; Miller et al. 2020; Merga and Van den Brink 2021; Macaulay et al. 2021). Major identified pathways include domestic wastewater contaminated via washing of pets, bedding, or owner hands, and direct entry into water bodies via treated pets swimming (Perkins et al. 2021; 2024; 2025; Yoder et al. 2024).

Our findings add a terrestrial dimension to these established aquatic contamination pathways. Many cavity-nesting birds, including blue tits and great tits, routinely incorporate mammalian fur into their nests. Our previous work (Tassin de Montaigu et al. 2025) found that 100% of tests nests contained fipronil residues and about 98% contained imidacloprid residues. Here, we demonstrate that concentrations in nest linings predict those in unhatched eggs and deceased chicks. This suggests that the compounds are being absorbed either via maternal transfer during egg formation or through dermal or oral exposure during early development. While our study did not quantify precise exposure pathways, several mechanisms may explain the observed transfer. Adults may have ingested the insecticides while building the nest, or while preening after laying in the nests, subsequently transferring residues to eggs (Kitulagodage et al. 2011; Ruuskanen et al. 2020), or to chicks via feeding (Grue et al. 1982; Poisson et al. 2021; Fuentes et al. 2025). Eggs may also absorb the compounds directly through the shell, particularly during the early stages of development when shells are more permeable (Stafford et al. 2018; Zhang et al. 2023; Wang et al. 2024). Chicks might have also directly ingested the compound and/or absorbed it dermally.

Imidacloprid and fipronil, together with their metabolites, were consistently detected in nest lining fur, likely reflecting differences in persistence, metabolism when compared to the other compounds screened. Fipronil’s lipophilicity favours accumulation in organic material such as fur, and imidacloprid, is environmentally persistent and repeatedly deposited. In contrast, rapidly metabolised compounds may be preferentially excreted and therefore remain undetectable in hair or fur matrices. Consequently, residues measured in nest material should not be interpreted as a direct quantitative proxy of exposure, as compound-specific metabolism and distribution strongly influence detectability.

While toxicity data for birds remains limited especially for smaller passerine species, the health implications of such exposure for birds are potentially severe. Fipronil and imidacloprid are known to disrupt neurological function (Eng et al. 2017; 2019), impair reproduction (Lopez-Antia et al. 2015a, b; Pandey and Mohanty 2015; Hussain et al. 2018), and alter behaviour in birds (Addy-Orduna et al. 2024). In addition, we found that the metabolite fipronil sulfone was found at a higher concentration in both unhatched eggs and dead chicks than the maternal compound fipronil. This could be explained by fipronil being transformed into fipronil sulfone in the body of the female with subsequent transfer into eggs (Zhang et al. 2023; Wang et al. 2024). Fipronil sulfone has been even less studied, but it has been suggested that the accumulation of the compound in brain tissues of quails (*Colinus virginianus*) induced reduced feed intake and high mortality (Kitulagodage et al. 2011; Gibbons et al. 2015). Given the neurotoxic mode of action of fipronil and imidacloprid, even low-level, chronic exposure during early development could lead to irreversible physiological or behavioural impairment in chicks. However, further experimental work is needed to determine threshold concentrations for adverse effects in passerine nestlings.

While our study provides foundational evidence for a novel exposure route, some limitations remain. Further controlled studies are needed to experimentally disentangle the transfer pathways (maternal transfer, ingestion, dermal absorption) and to assess dose–response relationships. We cannot yet distinguish the relative contribution of maternal versus postnatal transfer, nor determine whether exposure does harm. Furthermore, tissue samples were restricted to unhatched eggs and dead chicks, which may not capture the full spectrum of sub-lethal impacts occurring in live nestlings (e.g., compound found in specific organs). In addition, over 6 months occurred between the breeding season and the nests collection (from April to October), which likely allowed for the insecticides to degrade. Therefore, our data likely underestimates the concentrations that the eggs and chicks were exposed to during the nesting season.

Nonetheless, this study advances our understanding of how environmental contamination from domestic sources can directly expose wildlife. It highlights the need to consider the environmental safety of veterinary products and their potential impact on wildlife, as these treatments are applied frequently and year-round, which could contribute to a chronic and under-acknowledged source of chemical exposure.

Our work highlights that pesticide use can lead to unexpected pathways of environmental contamination. More specifically, this study adds to the weight of emerging evidence of widespread contamination (freshwater and birds’ nests) from pet parasiticides, such as fipronil and imidacloprid, showing an urgent need to re-evaluate the adequacy of current environmental risk assessments for companion animal veterinary drugs.

## Supplementary Information

Below is the link to the electronic supplementary material.

ESM 1 (DOCX 549 KB)

## Data Availability

The data that support the findings of this study are not openly available due to reasons of sensitivity and are available from the corresponding author upon reasonable request.
