# Supplementary material for: Transfer of veterinary parasiticides from the fur lining bird’s nest to eggs and chicks
Source: Environ Sci Pollut Res Int. 2026 Apr 21;33(15):7070–81. doi: 10.1007/s11356-026-37654-7 (PMC13156203; doi:10.1007/s11356-026-37654-7)
Supplement: Supplementary file 1 — (DOCX 549 KB) [file 11356_2026_37654_MOESM1_ESM.docx]

**Transfer of veterinary parasiticides from the fur lining bird’s nest to eggs and chicks**

Cannelle Tassin de Montaigu^a^, Gaetan Glauser^b^, Sylvie Guinchard^b^ & Dave Goulson^a^

^a^ School of Life Sciences, Department of Evolution, Behaviour & Environment, University of Sussex, Falmer, East Sussex, United Kingdom

^b^ Neuchâtel Platform of Analytical Chemistry, Faculty of Sciences, University of Neuchâtel, Neuchâtel, Switzerland

Corresponding author: Cannelle Tassin de Montaigu,

[ct430@sussex.ac.uk](mailto:ct430@sussex.ac.uk)

School of Life Sciences,

University of Sussex,

Falmer, East Sussex,

United Kingdom

S1: Questionnaire questions (and answer choices when relevant):

Is there farmland within 200m of your house? _____________________________

Circle if there are: cattle/sheep/goats/horses/poultry/pigs/dogs/cats

Do you have pets or other animals? ______________________ if yes,

What type e.g. dog/cat? ________________________________________

How many of each? __________________________________________

Do you treat them for fleas? _____________ if yes,

With what? _____________________________________________________

How frequent? _______________________________________________________

Please fill in the form as far as you can for each nest, leaving blank any questions you cannot answer

| Bag number | Date of nest collection | Location (postcode) | Habitat type (urban/rural) | species | Total number of eggs | Number of unhatched eggs | Total number of chicks | Number of dead chicks |
| --- | --- | --- | --- | --- | --- | --- | --- | --- |
|  |  |  |  |  |  |  |  |  |
|  |  |  |  |  |  |  |  |  |

S2: location of all the nests included in the study, coloured in relation to their urbanisation level (rural or urban) and sized in relation to their total concentration of compound (part per billion).


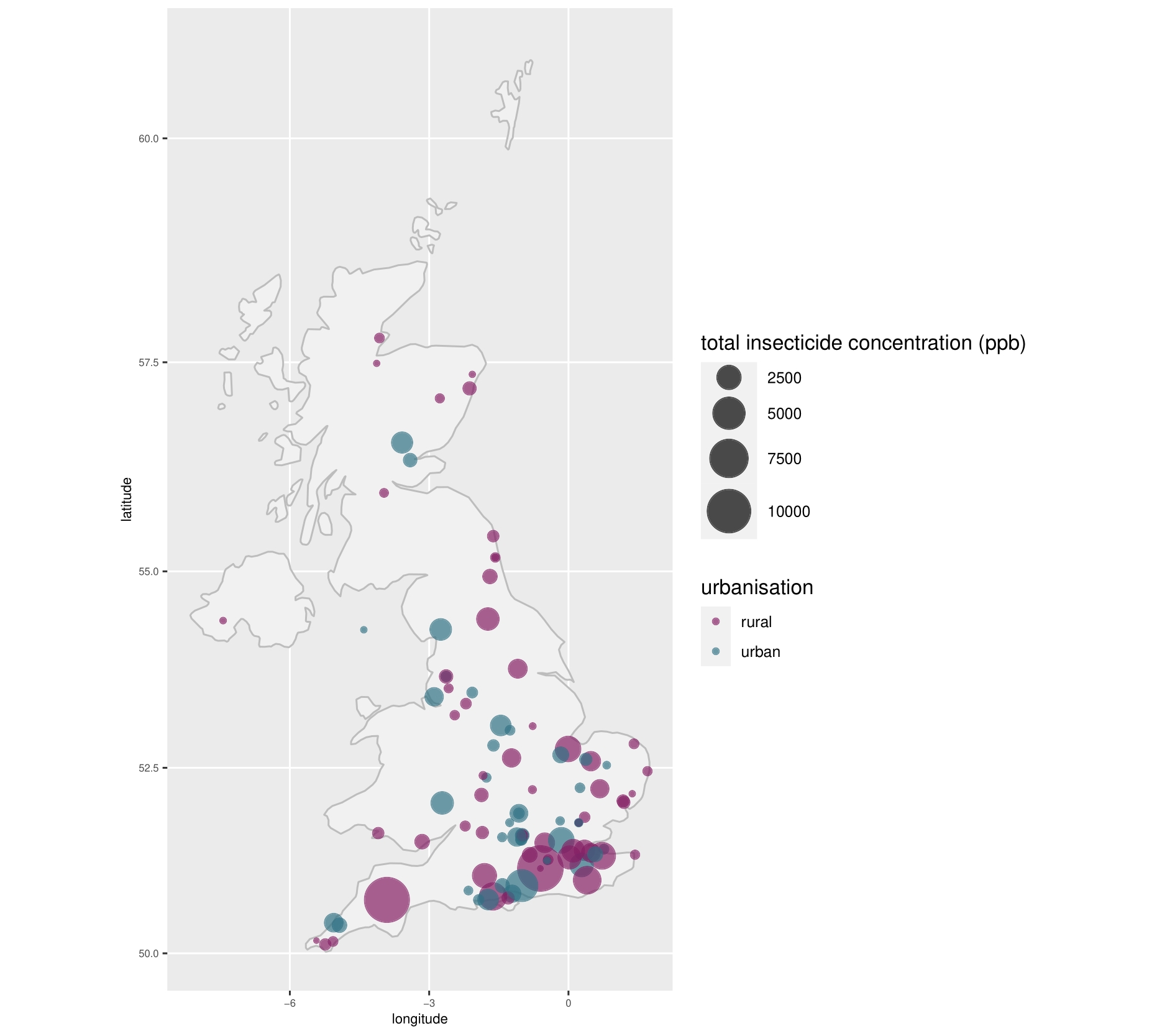


S3: list of pesticide screened for by HPLC-MS/MS, their respective pesticide class, substance type, LOQ (ppb) and LOD(ppb). * LOQ for chlorpyrifos and permethrin corresponds to 2x average peak in blanks due to some blank contamination, **desmethyl-acetamiprid LOQ and LOD are variable due to a strong interference in certain samples.

| **Pesticide** | **CAS number** | **Chemical formula** | **Molecular mass (g/mol)** | **Chemical class** | **Parasiticide type** | **LOQ (ng/g)** | **LOD (ng/g)** | **Chemical structure** |
| --- | --- | --- | --- | --- | --- | --- | --- | --- |
| Acetamiprid | 135410-20-7 | C_10_H_11_ClN_4_ | 222.67 | Neonicotinoid | Ectoparasiticide | 0.08 | 0.03 | 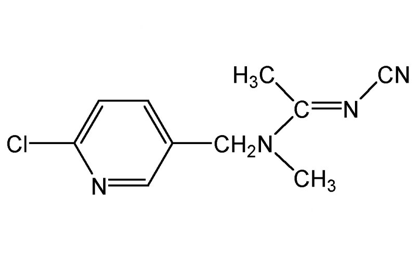 |
| Desmethyl-acetamiprid** | 190604-92-3 | C_9_H_9_ClN_4_ | 208.65 | Neonicotinoid | Ectoparasiticide | 0.08 - 8 | 0.03 - 2.67 | 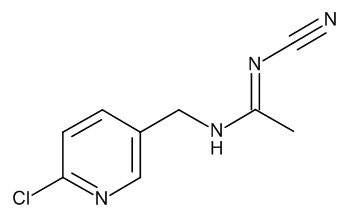 |
| Chlorpyrifos* | 2921-88-2 | C_9_H_11_Cl_3_NO_3_PS | 350.58 | Organophosphate | Ectoparasiticide | 0.8 | 0.27 | 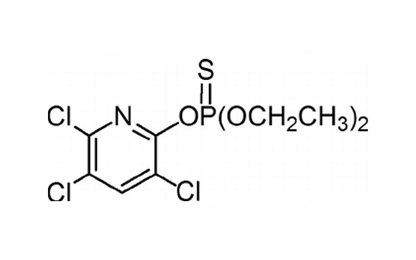 |
| Cypermethrin | 52315-07-8 | C_22_H_19_Cl_2_NO_3_ | 416.3 | Pyrethroid | Ectoparasiticide | 8 | 2.67 | 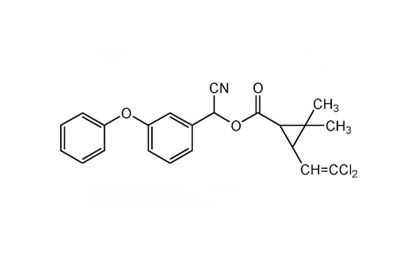 |
| Deltamethrin | 52918-63-5 | C₂₂H₁₉Br₂NO₃ | 505.2 | Pyrethroid | Ectoparasiticide | 1.6 | 0.53 | 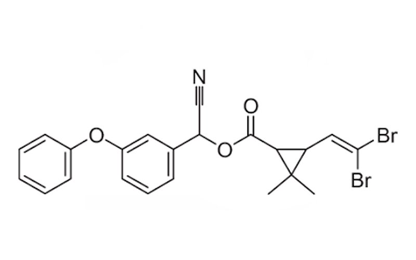 |
| Permethrin* | 52645-53-1 | C₂₁H₂₀Cl₂O₃ | 391.29 | Pyrethroid | Ectoparasiticide | 8 | 2.67 | 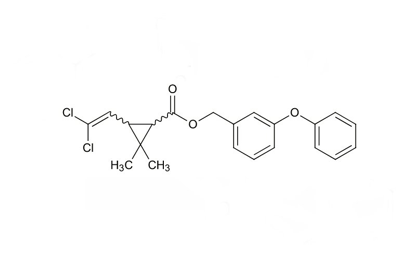 |
| Dinotefuran | 165252-70-0 | C₇H₁₄N₄O₃ | 202.21 | Neonicotinoid | Ectoparasiticide | 4 | 1.2 | 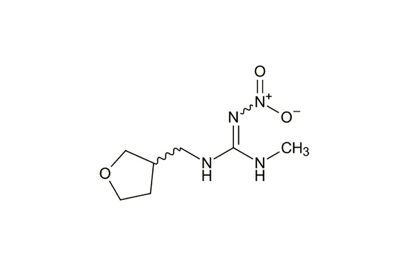 |
| Fipronil | 120068-37-3 | C₁₂H₄Cl₂F₆N₄OS | 437.15 | Phenylpyrazole | Ectoparasiticide | 0.16 | 0.05 | 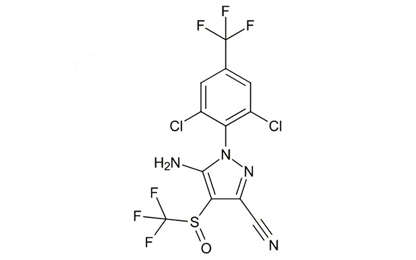 |
| Fipronil-sulfide | 120067-83-6 | C_12_H_4_Cl_2_F_6_N_4_S | 421.15 | Phenylpyrazole | Ectoparasiticide | 0.16 | 0.05 | 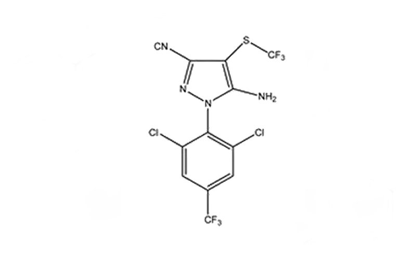 |
| Fipronil-sulfone | 120068-36-2 | C_12_H_4_Cl_2_F_6_N_4_O_2_S | 453.15 | Phenylpyrazole | Ectoparasiticide | 0.16 | 0.05 | 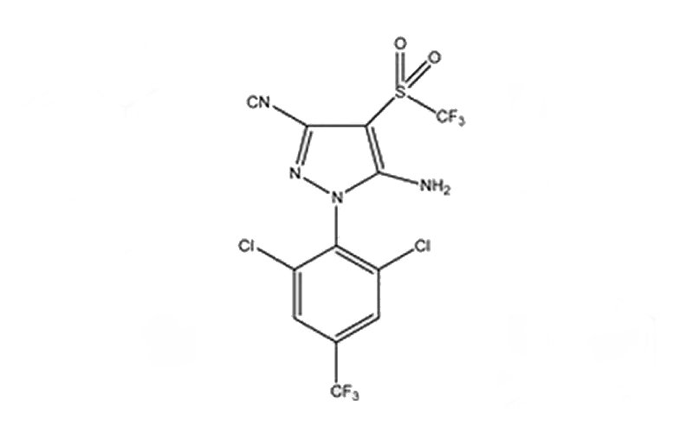 |
| Imidacloprid | 138261-41-3 | C₉H₁₀ClN₅O₂ | 255.66 | Neonicotinoid | Ectoparasiticide | 0.4 | 0.13 | 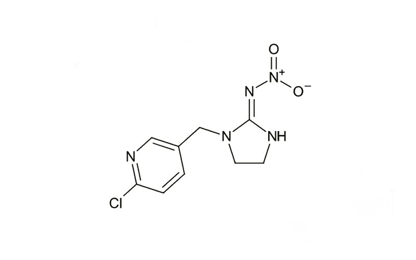 |
| Desnitroimidacloprid | 187022-17-9 | C_9_H_11_ClN_4_ | 210.66 | Neonicotinoid | Ectoparasiticide | 0.16 | 0.05 | 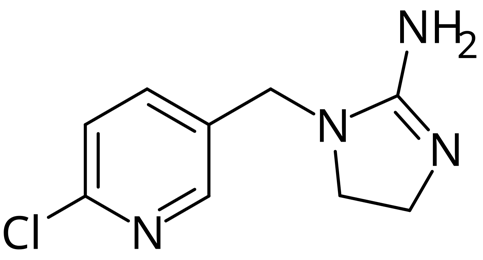 |
| Imidacloprid-olefin | 115086-54-9 | C_9_H_8_ClN_5_O_2_ | 253.64 | Neonicotinoid | Ectoparasiticide | 0.8 | 0.27 | 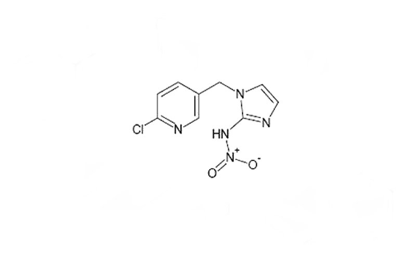 |
| Ivermectin | 70288-86-7 | C₄₈H₇₄O₁₄ + C₄₇H₇₂O₁₄ | 861.1 | Avermectin | Ectoparasiticide & endoparasiticide | 1.6 | 0.53 | 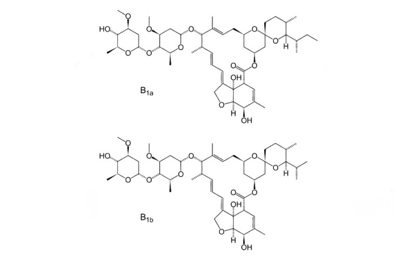 |
| Thiacloprid | 111988-49-9 | C₁₀H₉ClN₄S | 252.72 | Neonicotinoid | Ectoparasiticide & endoparasiticide | 0.08 | 0.03 | 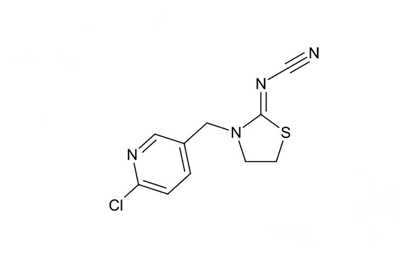 |
